# Distribution and dissemination of antimicrobial-resistant *Salmonella* in broiler farms with or without enrofloxacin use

**DOI:** 10.1186/s12917-018-1590-1

**Published:** 2018-08-30

**Authors:** Ke Shang, Bai Wei, Min Kang

**Affiliations:** Department of Veterinary Infectious Diseases and Avian Diseases, College of Veterinary Medicine and Center for Poultry Diseases Control, Chonbuk National University, 79 Gobong-ro, Iksan, 54596 South Korea

**Keywords:** *Salmonella*, Antimicrobial resistance, Circulating contamination, Enrofloxacin, Broiler farm, Litter, PFGE

## Abstract

**Background:**

*Salmonella* is a major zoonotic food-borne pathogen that persists on poultry farms, and animals undergo reinfection with endemic strains. The present study aimed to investigate the characteristics and dissemination of antimicrobial-resistant *Salmonella* within and between broiler farms that used enrofloxacin and those that did not.

**Results:**

Cloacal and environmental (litter, feed, and water) samples from two selected flocks in each of 12 farms owned by the same company were collected three times over a 30-day period of two production cycles during 2015–2016. The rate of *Salmonella* isolation was 7.8% (123/1584). Nine *Salmonella* serotypes (116 isolates) and seven untypable isolates were identified, and *Salmonella* Montevideo was the most prevalent serotype. Azithromycin-resistant (17.9%) and colistin-resistant (3.3%) isolates were detected, and multidrug-resistant isolates (43.1%) were also observed. No isolate was resistant to enrofloxacin or ciprofloxacin; however, intermediate resistance to enrofloxacin was significantly higher (*P* < 0.05) in farms that used enrofloxacin than in those that did not. The rate of multi-drug resistance among litter isolates (25/44, 56.8%) was significantly higher (*P* < 0.05) than that among cloacal swab (24/67, 35.8%) and feed (4/12, 33.3%) isolates. Pulsed-field gel electrophoresis (PFGE) analysis of strains of the same serotype was conducted to determine their epidemiological relationship. The PFGE types were classified into 31 groups with a 100% correlation cutoff in dendrograms for *Salmonella* Montevideo isolates, which showed 100% genomic identity based on age, sample type, flock, and production cycle within and between farms.

**Conclusion:**

The present study highlights the occurrence of horizontal transmission and cyclic contamination with antimicrobial-resistant *Salmonella* in broiler farms owned by the same company. Litter may be a good indicator of indoor environmental contamination with antimicrobial-resistant *Salmonella* on farms. Additionally, enrofloxacin use may be one of the factors promoting resistance towards it in *Salmonella*.

**Electronic supplementary material:**

The online version of this article (10.1186/s12917-018-1590-1) contains supplementary material, which is available to authorized users.

## Background

*Salmonella* is the leading cause of food-borne illness worldwide, and dissemination of antimicrobial-resistant *Salmonella* through the food chain, especially through chicken, has important implications for the failure of salmonellosis treatment. There is increasing risk of chicken-mediated spread of antimicrobial-resistant *Salmonella* to public health [1]. The prevalence of *Salmonella* on farms is linked to the prevalence of *Salmonella* in the derived meat products [2]. Prevention of *Salmonella* contamination in chicken products requires detailed knowledge of the major sources of contamination. Although measures for eliminating *Salmonella* on breeder farms through vaccination, use of all-in/all-out replacement systems on broiler farms, and “antimicrobial-free” strategies have been implemented, high *Salmonella* prevalence rates and antimicrobial-resistance rates are still observed in broiler farms [3, 4]. Interventions performed at poultry farms, including enhanced biosecurity, rodent control, routine surveillance of the farm environment, feed decontamination, and use of autogenous poultry vaccine, can reduce, but cannot eliminate *Salmonella* from live poultry [5]. Environmental samples, especially poultry litter, have been reported to be a good indicator for the presence of *Salmonella* in poultry farms [6, 7]. We speculated that the transmission of antimicrobial-resistant *Salmonella* among farms might occur through indoor environmental contamination in farms.

*Salmonella* strains can develop antimicrobial resistance (AMR), initially to the traditional first-line drugs chloramphenicol, ampicillin, and trimethoprim/sulfamethoxazole. Because of such resistance, fluoroquinolones (FQs), third-generation cephalosporins, and macrolides (azithromycin) have become critically important for treating salmonellosis in humans [8]. Prophylactic treatment of poultry with enrofloxacin (ENR), a fluoroquinolone antibiotic used to treat animal infections, has been implicated in the increasing resistance to ciprofloxacin, posing a risk to human health [9–11]. In Korea, ENR was licensed for veterinary use in 1987, and the quantity of ENR used has increased since its introduction [12]. In fact, the quantity of ENR sold was the highest of all antimicrobials used to treat chicken in Korea [13]. Concerns over the entry of antimicrobial-resistant zoonotic bacteria into the food chain and the consequent human infections led the Food and Drug Administration (FDA) to ban the use of ENR in poultry in the USA in September 2005 [14]. The use of antimicrobials in poultry farming and the accompanying selection pressure for resistant *Salmonella* have been the subjects of numerous studies [15, 16]. Antimicrobial use in farming has led to the widespread dissemination of antimicrobial-resistant *Salmonella* in broiler farms [4]. Therefore, to institute effective measures for reducing the infection of chicken with antimicrobial-resistant *Salmonella*, *Salmonella* contamination on broiler farms needs to be well-understood.

To explore production cycle-contamination with antimicrobial-resistant *Salmonella* in broiler farms that use antimicrobials and in those that do not, samples were collected from 12 farms (which either used or did not use ENR) owned by a single company during two production cycles. Trace-back investigations of the relationship between antimicrobial-resistant *Salmonella* and ENR use in broiler farms have not been previously conducted. The present study was aimed at determining the distribution and spread of indoor contamination with antimicrobial-resistant *Salmonella* through a comprehensive investigation of its prevalence within and between broiler farms. We also determined the epidemiological relationships among strains of the same serotype using electrophoresis-based DNA fingerprinting.

## Methods

### Sampling

The samples used in the present study were collected specifically for the purposes of the study. In total, 1584 fresh samples including 660 cloacal samples and 924 environmental samples (396 litter samples, 264 feed samples, and 264 water samples), were collected during 2015 and 2016 from 12 farms belonging to one of the largest integrated poultry companies in South Korea. In seven farms, ENR (50 mg/L) has been administered to young chicks via addition to drinking water for three consecutive days, while on the other five farms; no ENR was used [17]. The same ENR administration protocol was followed in all seven farms. The sampling farms contained an average of 70–100 thousands broilers, and 3–5 separate flocks. Two flocks per farm were sampled three times over a thirty-day period (chickens at 1, 15, and 30 days of age) during one production cycle. This sampling was then repeated during a second, separate production cycle. The cloacal samples were randomly collected from 25 broilers in entire area of the flock, and samples from five broilers were pooled into one test sample. The environmental samples, including litter (*n* = 15), feed (*n* = 10), and water (*n* = 10) were uniformly collected from equal areas of the flock, and five samples obtained from same area were pooled into one test sample. Finally cloacal swabs (S, *n* = 5), litter (L, *n* = 3), feed (F, *n* = 2) and water (W, *n* = 2) were collected from each flock.

### *Salmonella* isolation

Samples were collected in sterile plastic conical tubes (50 mL; SPL Life Sciences Co., Ltd., Seoul, Korea) and were stored under refrigeration in the laboratory until analysis, which was performed within 48 h of arrival. Fresh samples [1 g (or mL)] were separately mixed with 9 mL (1:9 dilutions) of buffered peptone water (BPW; BD Difco, Sparks, MD, USA) and incubated at 37 °C for 24 h for enrichment. An aliquot of the enriched BPW culture (100 μL) was transferred to 10 mL of Rappaport Vassiliadis (RV) broth (Thermo Fisher Scientific, Oxoid Ltd., Basingstoke, UK), and incubated at 42 °C for 24 h for selective enrichment [18]. A loopful of each RV culture was streaked onto a xylose-lysine-deoxycholate (XLD) agar plate (BD Difco™ XLD agar, USA), and the plate was incubated overnight at 37 °C. Presumptive *Salmonella* colonies were then tested with a *Salmonella* latex test kit (Thermo Fisher Scientific, Oxoid Ltd., Basingstoke, UK).

### *Salmonella* serotyping

The serogroup and serovar of each *Salmonella* isolate were identified following Edwards and Ewing’s procedure for the identification of *Enterobacteriaceae* using an antisera kit (BD Difco, Sparks, MD, USA) [19]. Somatic O antigen (BD Difco) was identified using the slide agglutination test with a commercially available antiserum. Flagellar (H) antigens (phases 1 and 2) were identified via successive inoculation onto 0.3% brain heart infusion (BHI) agar (BD) to activate flagella, followed by inoculation into BHI broth. The broth was cultured overnight, fixed with 0.6% formalin, and then analyzed using a tube agglutination test [20].

### Antimicrobial susceptibility

The minimum inhibitory concentrations (MICs) of the test antimicrobials nalidixic acid (NAL), ciprofloxacin (CIP), neomycin (NEO), gentamicin (GEN), streptomycin (STR), tetracycline (TET), azithromycin (AZM), amoxicillin/clavulanic acid (AMC), cefoxitin (FOX), ceftiofur (XNL), ampicillin (AMP), trimethoprim/sulfamethoxazole (SXT), colistin (COL), florfenicol (FFN), and chloramphenicol (CHL) were determined using the Sensititre panel KRNV4F (TREK Diagnostic Systems, Korea), while the MICs of enrofloxacin (ENR), tigecycline (TIG), and fosfomycin (FOS) were determined using the agar dilution method. *Escherichia coli* (ATCC 25922) were used as the quality-control strain. The susceptibility breakpoints of most tested antimicrobials were interpreted based on CLSI guidelines [21], while those of XNL, ENR, and FFN were interpreted based on CLSI BM31-A3 standards [22]. No CLSI interpretation criteria were available for STR, TIG, COL, and NEO; therefore, the following MIC values were considered to indicate resistance: STR, ≥ 64 μg/mL [23]; TIG, ≥ 8 μg/mL [24]; COL, ≥ 4 μg/mL [25]; and NEO, ≥ 16 μg/mL [26] (Table 1). *Salmonella* isolates resistant to at least three antimicrobial classes were designated multidrug resistant (MDR).

**Table 1:** Antimicrobials used in the study and the tested concentration ranges

Antimicrobials^a^	Abbreviation	Breakpoints (μg/mL)			Concentration ranges (μg/mL)
		S^b^	I	R	
Nalidixic acid	NAL	16	-^c^	32	2–128
Ciprofloxacin	CIP	0.06	0.12–0.5	1	0.12–16
Enrofloxacin	ENR	0.25	0.5–1	2	0.12–64
Neomycin	NEO	–	–	16	2–32
Gentamicin	GEN	4	8	16	1–64
Streptomycin	STR	32	–	64	2–128
Tetracycline	TET	4	8	16	2–128
Azithromycin	AZM	16	–	32	0.25–64
Amoxicillin/clavulanic acid	AMC	8/4	16/8	32/16	2/1–64/32
Cefoxitin	FOX	8	16	32	1–32
Ceftiofur	XNL	2	4	8	0.5–8
Ampicillin	AMP	8	16	32	2–32
Trimethoprim/sulfamethoxazole	SXT	2/38	–	4/76	0.12/2.38–4/76
Colistin	COL	2	–	4	2–32
Florfenicol	FFN	–	–	32	2–64
Chloramphenicol	CHL	8	16	32	2–64
Tigecycline	TIG	2	4	8	0.12–32
Fosfomycin	FOS	64	128	256	0.25–256

^a^Sorted based on category of antimicrobials

^b^S, sensitivity; I, intermediate resistance; R, resistance

^c^-, no standard breakpoint value in related references

### PFGE and BioNumerics analysis

Isolates of *Salmonella* Montevideo (*n* = 75) and *Salmonella* Senftenberg (*n* = 16) were genotyped using PFGE following protocols of the Centers for Disease Control and Prevention available on PulseNet, with some modifications. In brief, the *Salmonella* isolates were streaked onto MacConkey agar plates and incubated overnight at 37 °C. Then, the bacteria were suspended in PBS at an OD value of 0.6–0.8. Genomic DNA (extraction using 1% SDS and 1 mg/mL proteinase K, Biosesang, Seoul, Korea) samples were digested with 50 U of *Xba*I (Thermo Fisher Scientific, Inchon, Korea) at 37 °C for 3 h. The digested DNA was separated by electrophoresis in 0.5 x TBE buffer at 14 °C for 18 h using a CHEF-DR^@^ electrophoresis system (Bio-Rad, Hercules, CA, USA). The pulse time was ramped from 2.16 to 63.8 s. *Salmonella* Braenderup H9812, which was included as a molecular weight standard, was processed with each batch of isolate. The gels were stained with ethidium bromide, and the DNA patterns were visualized on a UV transilluminator (Bio Doc-It Imaging System, Upland, CA, USA). The DNA fingerprints obtained with PFGE were analyzed using BioNumerics (version 5.10 for Windows). Dice similarity coefficients were calculated based on pairwise comparison of the PFGE types of the isolates. The isolates were considered to have closely related band patterns based on molecular typing when their PFGE types had dice similarity coefficients of 100%, and were clustered at the 90% similarity level. Band-matching settings, with an optimization of 1.0% and a position tolerance of 1.0%, were applied.

### Statistical analysis

The chi-square test was used to test for significant differences in the rates of *Salmonella* isolation, MDR prevalence, and AMR rates between farms that used ENR and those that did not. *P* values less than 0.05 were considered statistically significant. Analyses were performed using SPSS version 19.0 (IBM Co., Armonk, NY, USA).

## Results

### Prevalence and serovars of *Salmonella*

**Fig. 1:**
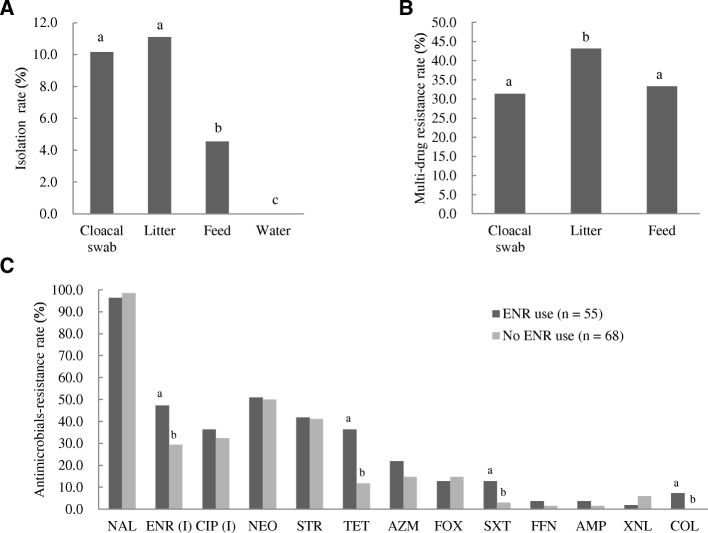
Rates of *Salmonella* isolation from samples of cloacal swabs, litter, feed, and water in broiler farms (**a**); the incidence of multi-drug resistance in isolates from cloacal swabs, litter, feed, and water in broiler farms (**b**); rates of antimicrobial-resistance in isolates from farms that used ENR and in isolates from farms that did not (**c**). The chi-square test was used to assess the significance of differences. *P* values less than 0.05 were considered statistically significant, and were marked with lowercase letters (a/b/c)

The cloacal and environmental samples (*n* = 1584) collected from the broiler farms were analyzed for the presence of *Salmonella*. In total, 123 (7.8%) samples were positive for *Salmonella*. There were no significant differences in isolation rates between the litter samples (44/396, 11.1%) and the cloacal swabs (67/660, 10.2%); however, significantly higher isolation rates were found in litter samples and cloacal swabs compared to feed (12/264, 4.5%), and water samples (0/264, 0.0%) (Fig. 1a). The *Salmonella* isolation rate from all types of samples was significantly lower (*P* < 0.05) in farms that used ENR (55/924, 6.0%) than on farms that did not (68/660, 10.3%; Table 2).

**Table 2:** *Salmonella* isolation from different sample types in broiler farms with or without enrofloxacin (ENR) use ^*^

ENR use	Sample no.	% (no. positive/total no. samples)				
		Cloacal swab	Litter	Feed	Water	Total
Yes	924	7.5 (29/385) ^a^	8.2 (19/231) ^a^	4.5 (7/154)	0.0 (0/154)	6.0 (55/924) ^a^
No	660	13.8 (38/275) ^b^	15.2 (25/165) ^b^	4.5 (5/110)	0.0 (0/110)	10.3 (68/660) ^b^
Total	1584	10.2 (67/660)	11.1 (44/396)	4.5 (12/264)	0.0 (0/264)	7.8 (123/1584)

^*^Lowercase (a/b) were used to indicate significant difference in isolation rates between farms with enrofloxacin use and those without; different letters indicate significant differences (*P* < 0.05)

Isolates were assigned to nine serovars, most of which belonged to serogroups C1 (65.0%) and E1 (17.1%). *S*. Montevideo (75 isolates, 61.0%) was the dominant serovar, and *S.* Senftenberg (21 isolates, 17.1%) was a distant second, followed by *S.* Emek (9 isolates, 7.3%), *S.* Macclesfield (4 isolate, 3.3%), and *S.* Virchow (3 isolates, 2.4%). Only one isolate was detected for the four serotypes, *S.* Infantis, *S.* Edinburg, *S.* Hato, and *S.* Vellore, and seven isolates (5.7%) marked as *S*. spp. could not be assigned to specific serotypes (Table 3).

**Table 3:** Antimicrobial resistance in *Salmonella* isolates from broiler farms ^a^

Serovar (Serogroup)	N (%)	NAL		NEO		STR		TET		AZM		XNL		FOX		AMP		SXT		COL		FFN		> 1 agent	≥3 agents	≥5 classes
		MIC_50/90_	*n* (%)	MIC_50/90_	*n* (%)	MIC_50/90_	*n* (%)	MIC_50/90_	*n* (%)	MIC_50/90_	*n* (%)	MIC_50/90_	*n* (%)	MIC_50/90_	*n* (%)	MIC_50/90_	*n* (%)	MIC_50/90_	*n* (%)	MIC_50/90_	*n* (%)	MIC_50/90_	*n* (%)	*n* (%)	*n* (%)	*n* (%)
Hato (B)	1 (0.8)	–	1 (100)	–	1 (100)	–	1 (100)	–	0	–	0	–	0	–	0	–	0	–	0	–	0	–	0	1 (100)	1 (100)	0
Vellore (B)	1 (0.8)	–	0	–	1 (100)	–	0	–	0	–	0	–	0	–	0	–	0	–	0	–	0	–	0	1 (100)	0	0
Montevideo (C1)	75 (61.0)	128/128	75 (100)	4/32	35 (46.7)	32/128	37 (49.3)	4/16	24 (32)	16/32	12 (16)	1/1	3 (4.0)	4/16	9 (12.0)	4/8	2 (2.7)	0.5/1	8 (10.7)	2/2	2 (2.7)	8/8	3 (4.0)	75 (100)	37 (49.3)	4 (5.3)
Virchow (C1)	3 (2.4)	128/128	3 (100)	16/32	3 (100)	64/64	2 (66.7)	8/8	0	64/64	2 (66.7)	1/1	0	16/32	1 (33.3)	8/8	0	0.25/0.25	0	2/2	0	8/8	0	3 (100)	2 (66.7)	0
Infantis (C1)	1 (0.8)	–	1 (100)	–	1 (100)	–	1 (100)	–	1 (100)	–	1 (100)	–	1 (100)	–	0	–	0	–	0	–	1 (100)	–	0	1 (100)	1 (100)	1 (100)
Edinburg (C1)	1 (0.8)	–	1 (100)	–	0	–	0	–	0	–	0	–	0	–	1 (100)	–	0	–	0	–	0	–	0	1 (100)	0	0
Emek (C2-C3)	9 (7.3)	128/128	9 (100)	4/16	3 (33.3)	8/32	1 (11.1)	1/4	0	16/32	3 (33.3)	0.5/1	0	4/16	0	2/4	0	0.5/0.5	0	2/2	0	4/8	0	9 (100)	3 (33.3)	0
Macclesfield (D2)	4 (3.3)	64/128	4 (100)	2/16	1 (25)	8/128	1 (25)	1/16	1 (6.3)	16/64	1 (25)	0.5/1	0	4/32	0	2/8	0	0.5/4	1 (25)	2/2	0	2/8	0	4 (100)	1 (25)	0
Senftenberg (E4)	21 (17.1)	128/128	21 (100)	4/32	10 (47.6)	32/128	4 (19)	1/4	0	16/16	2 (9.5)	0.5/1	0	4/16	2 (9.5)	2/4	0	0.5/0.5	0	2/2	0	4/8	0	21 (100)	2 (9.5)	0
spp. (C2-C3, B, D1)	7 (5.7)	–	6 (71.4)	–	7 (100)	–	4 (57.1)	–	2 (28.6)	–	1 (14.3)	–	1 (14.3)	–	4 (57.1)	–	1 (14.3)	–	0	–	1 (14.3)	–	0	7 (100)	4 (57.1)	2 (28.6)
Total	123	128/128	120 (97.6)	8/32	62 (50.4)	8/32	51 (41.5)	1/16	28 (22.8)	16/32	22 (17.9)	1/1	5 (4.1)	4/32	17 (13.8)	4/8	3 (2.4)	0.5/0.5	9 (7.3)	2/2	4 (3.3)	8/8	3 (2.4)	123 (100)	53 (43.1)	7 (5.7)

^a^-, not analyzed; the unit of MIC_50/90_ was μg/mL

### Antimicrobial susceptibility analysis

All the isolates analyzed in this study showed resistance to at least one tested antimicrobial (Table 3). Resistance to NAL was the most prevalent (120/123, 97.6%), followed by that to NEO (62/123, 50.4%), STR (51/123, 41.5%), TET (28/123, 22.8%), AZM (22/123, 17.9%), FOX (17/123, 13.8%), XNL (5/123, 4.1%), AMP (3/123, 2.4%), SXT (9/123, 7.3%), COL (4/123, 3.3%), and FFN (3/123, 2.4%). several isolates showed intermediate resistance (IR) to ENR (46/123, 37.4%) and CIP (42/123, 31.1%). All isolates were sensitive to the five antimicrobials CHL, GEN, TIG, FOS, and AMC.

Thirty-one AMR phenotypes were observed among the *Salmonella* isolates from the 12 broiler farms; the AMR profile NAL + NEO + STR + TET was the most prevalent in this study (10 isolates, 8.1%) (Table 4). Further, seven MDR isolates comprising four *S*. Montevideo, two *S*. spp., and one *S*. Infantis isolates, were resistant to ≥5 antimicrobial classes, all of which were isolated from the farms that used ENR.

**Table 4:** Antimicrobial resistance profiles of *Salmonella* isolates from broiler farms (*n* = 123)

No.	Antimicrobial resistance profile	*n*^*a*^ (%)	Serovars (*n*)
–	Susceptible	0	–
1	NAL	27 (22.0)	Montevideo (13), Senftenberg (8), Emek (5), Macclesfield (1)
2	NEO	2 (1.6)	Vellore (1), spp. (1)
3	NAL + NEO	18 (14.6)	Montevideo (9), Senftenberg (6), Virchow (1), spp. (2)
4	NAL + STR	6 (4.9)	Montevideo (5), Senftenberg (1)
5	NAL + AZM	4 (3.3)	Montevideo (1), Senftenberg (1), Emek (1), Macclesfield (1)
6	NAL + FOX	3 (2.4)	Montevideo (1), Senftenberg (1), Edinburg (1)
7	NAL + SXT	9 (7.3)	Montevideo (8), Macclesfield (1)
8	NAL + COL	1 (0.8)	Montevideo (1)
9	NAL + NEO + STR	7 (5.7)	Montevideo (3), Senftenberg (2), Emek (1), Hato (1)
10	NAL + NEO + FFN	2 (1.6)	Montevideo (2)
11	NAL + NEO + AZM	4 (3.3)	Montevideo (1), Senftenberg (1), Emek (2)
12	NAL + NEO + COL	1 (0.8)	Montevideo (1)
13	NEO + STR + FOX	3 (2.4)	Montevideo (3)
14	NEO + STR + TET	4 (3.3)	Montevideo (4)
15	NAL + STR + AZM	2 (1.6)	Montevideo (2)
16	NEO + STR + TET + FOX	1 (0.8)	spp. (1)
17	NAL + STR + FOX + AZM	1 (0.8)	Montevideo (1)
18	NAL + NEO + STR + TET	10 (8.1)	Montevideo (10)
19	NAL + NEO + STR + FOX	2 (1.6)	Senftenberg (1), spp. (1)
20	NAL + NEO + STR + AZM	1 (0.8)	Virchow (1)
21	NAL + NEO + FFN + AZM	1 (0.8)	Montevideo (1)
22	NAL + NEO + STR + TET + XNL	2 (1.6)	Montevideo (2)
23	NAL + NEO + STR + TET + FOX	2 (1.6)	Montevideo (1), Macclesfield (1)
24	NAL + NEO + STR + TET + AZM	2 (1.6)	Montevideo (2)
25	NAL + NEO + STR + FOX + AZM	1 (0.8)	Virchow (1)
26	NAL + NEO + STR + FOX + COL + AZM	1 (0.8)	spp. (1)
27	NAL + NEO + STR + TET + FOX + AZM	2 (1.6)	Montevideo (2)
28	NAL + STR + TET + FFN + AMP + AZM	1 (0.8)	Montevideo (1)
29	NAL + NEO + STR + TET + FOX + XNL + AMP	1 (0.8)	spp. (1)
30	NAL + NEO + STR + TET + XNL + COL + AZM	1 (0.8)	Infantis (1)
31	NAL + NEO + STR + TET + FOX + XNL + AMP + AZM	1 (0.8)	Montevideo (1)

^a^*n*, number of isolates

The MDR rate among litter isolates (25/44, 56.8%) was significantly higher (*P* < 0.05) than that among the cloacal swab (24/67, 35.8%) and feed (4/12, 33.3%) isolates (Fig. 1b). The ratio of IR to ENR was significantly higher (*P* < 0.05) in isolates from farms that used ENR (26/55, 47.3%) than in those from farms that did not (20/68, 29.4%; Fig. 1c).

### Serotype distribution and genetic analysis of isolates from broiler farms

**Fig. 2:**
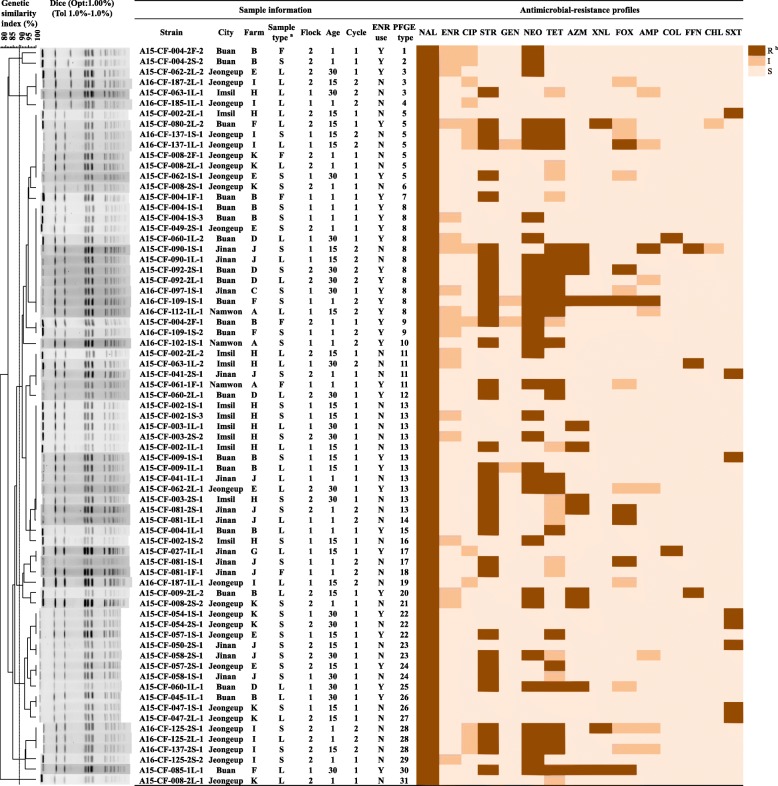
Dendrograms showing pattern analysis on the basis of *Xba* I-PFGE of the 75 *Salmonella* Montevideo isolates obtained from broiler farms, along with related sampling information, and antimicrobial-resistance (AMR) profiles. The Dice coefficient was used to perform similarity analysis. ^a^S, cloacal swabs; L, litter; F, feed. ^b^R, resistance (dark pink); I, intermediate resistance (pink), S, sensitivity (light pink). Dotted lines indicate 90% similarity

Serotype diversity was observed in the broiler farms sampled in the present study with 75% (9/12) of them showing contamination with at least two *Salmonella* serovars (Table 5). *S.* Montevideo and *S.* Senftenberg were isolated from 11 and 8 of the 12 farms, respectively. In farms B, H, I, and J, there were at least four different serotypes observed in a single flock. In one farm (farm H), isolates from the same flock at different ages had the same PFGE type (type 13), with a similarity index of 100% (isolates A15-CF-002-1S-3 and A15-CF-003-1 L-1); isolates from different flocks were of the same type (type 13; isolates A15-CF-003-1 L-1 and A15-CF-003-2S-2); and two isolates, A15-CF-002-2 L-2 and A15-CF-063-1 L-2, which were of the same PFGE type (type 11) and same AMR profile (including intermediate ENR resistance), were obtained from litter during different production cycles. Isolates of the same PFGE type were also observed in different farms (type 5 in farms H, F, I, K, and E; type 8 in farms B, E, D, J, C, F, and A; type 11 in farms A, H, and J; type 13 in farms B, E, H, and J; type 17 in farms G and J; type 22 in farms K and E; and type 26 in farms B and K; Fig. 2). In *S.* Senftenberg isolates, the same PFGE type was observed in two different farms in different cities, that neither of which used ENR (type 4 in farms H and J; type 6 in farms J and K; Additional file 1).

**Table 5:** Distribution of *Salmonella* serotypes and genotypes in broiler farms

Farm	ENR use ^a^	Flock	Production cycle 1			Production cycle 2		
			1 day	15 days	30 days	1 day	15 days	30 days
A	Y	1	*S*. Montevideo (11)^b^ *S*. Senftenberg (5)	-^c^	–	*S*. Montevideo (10) *S*. Macclesfield (NC^d^)	*S*. Montevideo (8)	–
		2	–	–	–	–	–	*S*. spp. (NC) *S*. Vellore (NC)
B		1	*S*. Montevideo (7, 8, 15)	*S*. Montevideo (13)	*S*. Montevideo (26)	*S*. Senftenberg (7)	–	–
		2	*S*. Montevideo (2, 4) *S*. Senftenberg (10)	*S*. Montevideo (20) *S*. Emek (3)	*S.* Macclesfield (NC)	–	–	–
C		1	–	–	*S*. Montevideo (8)	*S*. Montevideo (28) *S*. Senftenberg (9) *S*. Emek (4)	–	–
		2	–	–	–	*S*. spp. (NC)	–	–
D		1	*S*. Senftenberg (2)	*S*. Montevideo (8)	*S*. Montevideo (8, 25)	–	–	–
		2	*S*. Macclesfield (NC)	*S*. Montevideo (8)	*S*. Montevideo (12)	–	–	*S*. Montevideo (8)
E		1	–	*S*. Montevideo (22)	*S*. Montevideo (5)	–	–	–
		2	*S*. Montevideo (8)	*S*. Montevideo (24)	*S*. Montevideo (3, 13)	–	–	–
F		1	–	*S*. Virchow (NC)	*S*. Montevideo (30) *S*. spp. (NC)	*S*. Montevideo (8, 9)	–	–
		2	–	*S*. Montevideo (5) *S*. Virchow (NC)	*S*. Infantis (NC)	–	–	–
G		1	–	*S*. Montevideo (17)	–	N^e^	N	N
		2	–	–	–	N	N	N
H	N	1	*S*. Senftenberg (11) *S*. Virchow (NC)	*S*. Montevideo (13, 16) *S*. Senftenberg (4)	*S*. Montevideo (13)	*S*. Macclesfield (NC)	*S*. Montevideo (13)	*S*. Montevideo (3, 11)
		2	*S*. Senftenberg (8)	*S*. Montevideo (5, 11)	*S*. Montevideo (13)	–	–	–
I		1	–	*S*. Montevideo (19)	–	*S*. Montevideo (4) *S*. Senftenberg (8) *S*. Emek (2)	*S*. Montevideo (5) *S*. Edinburg (NC)	–
		2	*S*. Montevideo (29)	*S*. Montevideo (28) *S*. Hato (NC)	–	*S*. Montevideo (28) *S*. Senftenberg (2)	*S*. Montevideo (16, 28) *S*. Senftenberg (2)	*S*. Montevideo (28)
J		1	*S*. Montevideo (13) *S*. Emek (1,2)	*S*. spp. (NC)	*S*. Montevideo (24)	*S*. Montevideo (14, 17, 18) *S*. Senftenberg (4)	*S*. Montevideo (8)	–
		2	*S*. Montevideo (11)	*S*. Montevideo (23)	*S*. Montevideo (23)	*S*. Montevideo (13) *S*. Senftenberg (4)	*S*. Montevideo (14) *S*. Senftenberg (6)	–
K		1	*S*. Senftenberg (6)	*S*. Montevideo (26)	*S*. Montevideo (22)	–	–	–
		2	*S*. Montevideo (5, 6, 21, 31)	*S*. Montevideo (27)	*S*. Montevideo (22)	–	–	*S*. spp. (NC)
L		1	*S*. Senftenberg (NC)	*S*. Senftenberg (NC)	–	N	N	N
		2	–	–	–	N	N	N

^a^ENR use, Y implies yes; N implies no.

^b^PFGE types are showed within parenthesis

^c^The dash (−) indicates that the farm was negative for *Salmonella*

^d^NC implies that PFGE was not conducted

^e^N denotes no sampling

## Discussion

In the present study, to explore the distribution and dissemination of antimicrobial-resistant *Salmonella* in broiler farms, we obtained samples during two production cycles from farms that used antimicrobials and those that did not (Table 5).

*S*. Montevideo, one of the serotypes prevalent in human infections [27], showed high resistance to ≥3 antimicrobials (49.3%; Table 3). In recent years, there has been an increase in *S*. Montevideo human infections and outbreaks worldwide, including in the USA, Europe, Australia, and Asian nations, such as South Korea [5, 28–30]. In the USA, the number of human illnesses caused by *S*. Montevideo increased from 728 cases in 2002 to 1203 cases in 2012 [31]. In South Korea, *S*. Montevideo is one of the most common serotypes affecting the poultry industry [32, 33]. Additionally, the most serious case of resistance found in the present study was an *S*. Montevideo isolate with resistance to eight antimicrobials (NAL + NEO + STR + TET + FOX + XNL + AMP + AZM; Table 4). In addition, there was an MDR *S*. Infantis isolate resistant to ≥5 antimicrobial classes (Table 3); and *S*. Infantis is one of the most prevalent serotypes in South Korea and second in frequency among serovars [34]. In countries including Germany [35], Hungary [36], Italy [37], and Japan [38], emergence and clonal dissemination of MDR *S*. Infantis strains in chicken has been recent reported, and has been related to an increased incidence of human infections [31]. Given these findings, further investigations of the antimicrobial resistance and distribution of *S*. Infantis in slaughterhouses and retail chicken meat are required.

All isolates showed resistance to at least one antimicrobial, suggesting that AMR *Salmonella* is widespread in broiler farms, as was reported previously in South Korea [33]. Contrary to the increasing prevalence of FQ-resistant *Salmonella* reported in other countries [39, 40], we did not observe any FQ-resistant isolates in this study; however, 31.1% of the isolates showed reduced susceptibility to CIP (with a MIC of 0.12 to 0.25 or 0.5 μg/mL), and 37.4% of the isolates showed IR to ENR [41, 42]. The rate of IR to CIP was higher in farms that used ENR than in farms that did not, although the difference was not statistically significant (*p* > 0.05). The ratio of IR to ENR was significantly higher (*P* < 0.05) in farms that used ENR than in those that did not (Fig. 1c). Resistance to SXT, TET, and COL was significantly higher in farms that used ENR than in those that did not (*P* < 0.05; Fig. 1c). Resistance to the three antimicrobials ENR, TET, and SXT was most commonly observed in isolates from chicken, in a previous study [43]. One plausible explanation is that the selective pressure exerted by the antimicrobials contributed to the co-selection of this antimicrobial resistance pattern. The co-selection of resistance to more than one antimicrobial, owing to the genetic linkage of resistance genes, is a common feature of resistance acquired by horizontal gene transfer [44, 45].

In the present study, 17.9% of isolates showed resistance to AZM (Table 3), a macrolide antimicrobial used for the limited treatment of MDR *Salmonella* infections [8, 46]. In recent years, AZM has been used for typhoid treatment due to the increased prevalence of MDR *Salmonella* isolates, which has caused serious problems in antimicrobial selection. The sporadic occurrence of AZM-resistant *Salmonella* isolates has also caused problems in the selection of suitable antimicrobials for typhoid treatment [47, 48]. In Europe, the first case of failure of AZM treatment of *Salmonella* infection was reported [49]. Similarly, emergence of clinical AZM-resistant *Salmonella* isolates was recently reported in Asia [50, 51]; 15.24% of *Salmonella* isolates from chicken showed resistance to AZM (with an MIC ≥16 μg/mL) in another study [52]. However, reports of AZM resistance in *Salmonella* isolates from humans or animals in South Korea are rare. Considering the fact that, AZM has not been used on these farms to the best of our knowledge, the prevalence of AZM resistance in *Salmonella* is probably either due to prolonged use of antimicrobials in the same class as AZM such as erythromycin and tylosin for treating poultry diseases [53], or due to co-resistance to AZM and other antimicrobials [54]. Further, the MIC_50_ of AZM was 16 μg/mL against all isolates and 64 μg/mL against *S*. Virchow (Table 3). However, the exact reason for the emergence of AZM resistance in *Salmonella* and its mechanism require further studies. Additionally, because COL is a last-line drug used to treat MDR *Enterobacteriaceae* infections, the four COL-resistant isolates identified in the present study warrant more attention; due to the prevalence of COL-resistant *Salmonella*, *mcr* family genes can be easily and quickly transmitted [8, 55, 56]. In Europe, plasmid-mediated COL resistance in *Enterobacteriaceae* has already spread widely in avian and pig farms, and this has necessitated prompt international action to restrict or ban COL use in agriculture to avoid further spread of resistance, similar to the solution involving NDM-1 (New Delhi metallo-β-lactamase-1) several years ago [56]. COL resistance in *Salmonella* isolates from humans had not been reported until recently, when the first COL-resistant isolates from humans were reported in the Arabian Peninsula [57], followed by two COL-resistant *Salmonella* strains in Italy [58]. The observation of co-resistance to AZM and COL in two MDR isolates (AMR profiles: NAL + NEO + STR + FOX + COL + AZM and NAL + NEO + STR + TET + XNL + COL + AZM), both of which exhibited intermediate resistance to CIP and ENR (data not shown), is of high importance. This might pose a considerable challenge when selecting drugs to treat human *Salmonella* infections. The most important finding of the present study was that the AMR profile XNL + COL + AZM, conferring resistance to the critically important antimicrobials used to treat salmonellosis or MDR *Salmonella* infections in humans, has never been reported previously in *Salmonella* strains of animal origin.

Based on the results of distribution of *Salmonella* serotypes and genotypes, a considerable cross-contamination among the farms could be inferred. For example, isolates with the same PFGE type shared between flocks, production cycles, sample types, and among chickens of different ages within the farms were frequently identified (Fig. 2). This could be because the farms shared resources including breeders, trucks for transport, veterinarians, chicks, and feed [59]. Chicks in five farms (farms A, B, D, E, and J) were from the same breeder farm which was negative for *Salmonella* (data not shown). Moreover, there was contamination between flocks, production cycles, sample types, and among chicken of different ages within the farms. Antimicrobial-resistant *Salmonella* can be circularly transmitted between continuous production cycles. Although all-in/all-out replacement systems have been applied in the commercial poultry industry, *Salmonella* contamination still occurs, especially in the farm environment. Plausible explanations include insufficient disinfection, development of resistance to disinfectants in the first production cycle and subsequent survival, and inherent resistance in *Salmonella* [1, 60]. Another explanation is that other factors including air, unclean facilities, and vectors such as insects, wild birds, farmers, and rodents, might contribute to *Salmonella* transmission in poultry farms [61].

The dissemination of antimicrobial-resistant *Salmonella* in the farms might also have occurred via litter contamination (farm H; Fig. 2), because the litter samples had higher *Salmonella* isolation and MDR rates than the cloacal swab and feed samples (Fig. 1a, b), with no significant difference in isolation rates from litter samples between production cycles (Additional file 2). The spread of indoor *Salmonella* contamination in the broiler farms was apparently caused by litter from the broilers. *Salmonella*-positive litter samples detected in our study may have important public health implications. A recent study reported a positive correlation between prevalence of *Salmonella* in litter samples and *Salmonella* isolation from broiler carcasses [6]. Antimicrobial-resistant *Salmonella* could re-circulate in the farms because of litter contamination during different production cycles. Therefore, litter in broiler farms may be an important reservoir of *Salmonella*, consistent with a speculation in a report from the USA [62]. In summary, we concluded that serious *Salmonella* contamination occurs in farms during production cycles, as does cross-contamination among farms owned by the same company. Because of dissemination and cross-contamination between the farms that used antimicrobials and those that did not, close attention should be paid to farm-level hygiene management.

## Conclusion

In conclusion, we detected considerable contamination with antimicrobial-resistant *Salmonella* in broiler farms. The litter in the farm was one of the important reservoirs for *Salmonella* showing high *Salmonella* prevalence and MDR rates. Other reservoirs of *Salmonella*, such as feed, air, fans, and vectors such as insects, wild birds, farmers, and rodents might also contribute to its transmission in chicken farms [61]. Additional measures for litter and feed management might be required to prevent the transmission of antimicrobial-resistant *Salmonella* in such farms. Moreover, ENR use may be an important factor causing ENR resistance among *Salmonella* in the farms. Our results provide useful information regarding the distribution of AMR phenotypes among *Salmonella* isolates from broiler farms that use ENR and those that do not, highlighting the need for improved farming practices and more cautious use of antimicrobial agents. Further studies are required to develop protocols to prevent the contamination of litter and feed; this, together with instructions for strict all-in/all-out replacement and biosecurity systems, may markedly reduce the occurrence of antimicrobial-resistant *Salmonella* species in broiler farms.

## Additional files

Additional file 1: Dendrograms showing pattern analysis on the basis of *Xba* I-PFGE of the 16 *Salmonella* Senftenberg isolates obtained from broiler farms and their association with antimicrobial-resistance. The Dice coefficient was used to perform similarity analysis. ^a^S, cloacal swabs; L, litter; F, feed. ^b^R, resistance (dark pink); I, intermediate resistance (pink), S, sensitivity (light pink). Dotted lines indicate 90% similarity. (DOCX 271 kb)

Additional file 2: Isolation rates and multidrug resistant (MDR) rates in different types of samples obtained during production cycles 1 and 2. Different lowercase (a/b) and capital letters (A/B) in the same row were used to indicate significant (*P* < 0.05) differences in isolation rates and MDR rates between production cycles 1 and 2, respectively. (DOCX 36 kb)

## Abbreviations

AMC: Amoxicillin/clavulanic acid
AMP: Ampicillin
AMR: Antimicrobial resistance
AZM: Azithromycin
BHI: Brain heart infusion
CHL: Chloramphenicol
CIP: Ciprofloxacin
CLSI: Clinical Laboratory Standardization Institute
COL: Colistin
ENR: Enrofloxacin
FDA: Food and Drug Administration
FFN: Florfenicol
FOS: Fosfomycin
FOX: Cefoxitin
FQs: Fluoroquinolones
GEN: Gentamicin
MDR: Multidrug resistant
MICs: Minimum inhibitory concentrations
NAL: Nalidixic acid
NDM-1: New Delhi metallo-β-lactamase-1
NEO: Neomycin
PFGE: A pulsed-field gel electrophoresis
RV: Rappaport Vassiliadis
*S*. Edinburg: *Salmonella* Edinburg
*S*. Emek: *Salmonella* Emek
*S*. Hato: *Salmonella* Hato
*S*. Infantis: *Salmonella* Infantis
*S*. Macclesfield: *Salmonella* Macclesfield
*S*. Montevideo: *Salmonella* Montevideo
*S*. Senftenberg: *Salmonella* Senftenberg
*S*. spp.: *Salmonella* species
*S*. Vellore: *Salmonella* Vellore
*S*. Virchow: *Salmonella* Virchow
STR: Streptomycin
SXT: Trimethoprim/sulfamethoxazole
TET: Tetracycline
TIG: Tigecycline
USA: United States of America
XLD: Xylose-lysine-deoxycholate
XNL: Ceftiofur
