# Supplementary material for: Distribution and dissemination of antimicrobial-resistant Salmonella in broiler farms with or without enrofloxacin use
Source: BMC Vet Res. 2018 Aug 30;14:257. doi: 10.1186/s12917-018-1590-1 (PMC6117923; doi:10.1186/s12917-018-1590-1)

**Additional file 1:** Dendrograms showing pattern analysis on the basis of *Xba* I-PFGE of the 16 *Salmonella* Senftenberg isolates obtained from broiler farms and their association with antimicrobial-resistance. The Dice coefficient was used to perform similarity analysis. ^a^S, cloacal swabs; L, litter; F, feed. ^b^R, resistance (dark pink); I, intermediate resistance (pink), S, sensitivity (light pink). Dotted lines indicate 90% similarity.


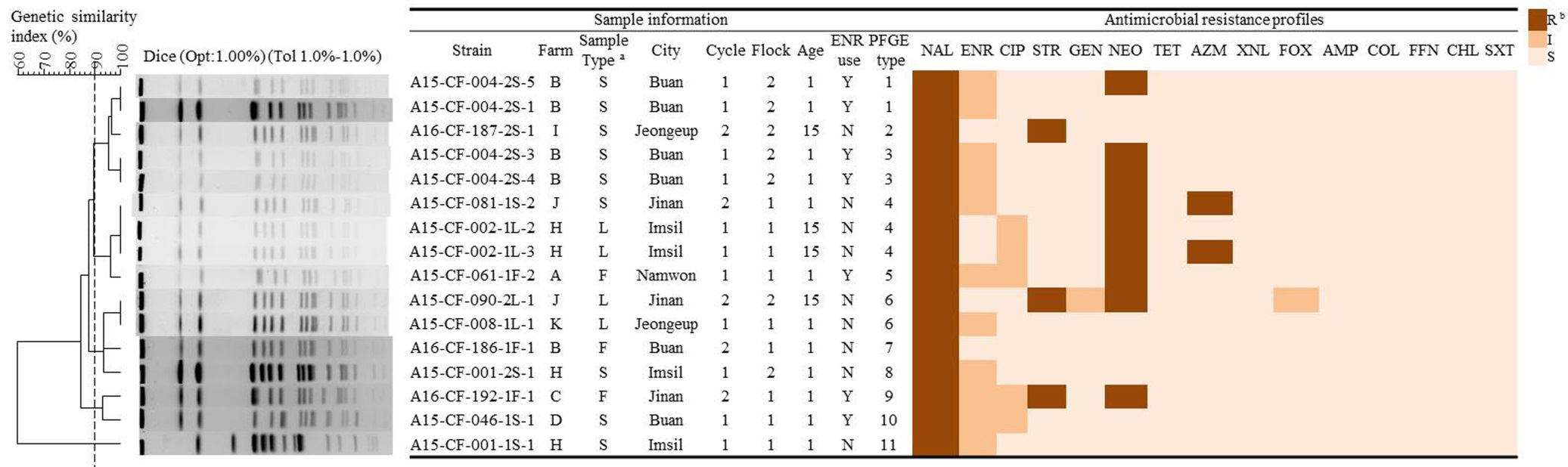

Supplement: Supplementary file 1 — Dendrograms showing pattern analysis on the basis of Xba I-PFGE of the 16 Salmonella Senftenberg isolates obtained from broiler farms and their association with antimicrobial-resistance. The Dice coefficient was used to perform similarity analysis. aS, cloacal swabs; L, litter; F, feed. bR, resistance (dark pink); I, intermediate resistance (pink), S, sensitivity (light pink). Dotted lines indicate 90% similarity. (DOCX 271 kb) [file 12917_2018_1590_MOESM1_ESM.docx]
