# Supplementary material for: Distribution and dissemination of antimicrobial-resistant Salmonella in broiler farms with or without enrofloxacin use
Source: BMC Vet Res. 2018 Aug 30;14:257. doi: 10.1186/s12917-018-1590-1 (PMC6117923; doi:10.1186/s12917-018-1590-1)
Supplement: Supplementary file 2 — Isolation rates and multidrug resistant (MDR) rates in different types of samples obtained during production cycles 1 and 2. Different lowercase (a/b) and capital letters (A/B) in the same row were used to indicate significant (P < 0.05) differences in isolation rates and MDR rates between production cycles 1 and 2, respectively. (DOCX 36 kb) [file 12917_2018_1590_MOESM2_ESM.docx]

**Additional file 2**: Isolation rates and multidrug resistant (MDR) rates in different types of samples obtained during production cycles 1 and 2^*^

| Sample types | Cycle 1 | | |  | Cycle 2 | | |  | Total | | |
| --- | --- | --- | --- | --- | --- | --- | --- | --- | --- | --- | --- |
|  | Sample no. | Isolate no. (%) | MDR isolate no. (%) |  | Sample no. | Isolate no. (%) | MDR isolate no. (%) |  | Sample no. | Isolate no. (%) | MDR isolate no. (%) |
| Cloacal swab | 360 | 49 (13.6) ^a^ | 14 (28.6) ^A^ |  | 300 | 18 (6.0) ^b^ | 10 (55.6) ^B^ |  | 660 | 67 (10.2) | 24 (35.8) |
| Litter | 216 | 26 (12.4) | 15 (57.7) |  | 180 | 18 (10.0) | 10 (55.6) |  | 396 | 44 (11.1) | 25 (56.8) |
| Feed | 144 | 6 (4.2) | 2 (33.3) |  | 120 | 6 (5.0) | 2 (33.3) |  | 264 | 12 (4.5) | 4 (33.3) |
| Water | 144 | 0 (0.0) | 0 (0.0) |  | 120 | 0 (0.0) | 0 (0.0) |  | 264 | 0 (0.0) | 0 (0.0) |
| Total | 864 | 81 (9.4) ^a^ | 31 (37.3) |  | 720 | 42 (5.8) ^b^ | 22 (55.0) |  | 1,584 | 123 (7.8) | 53 (43.1) |

^*^Different lowercase (a/b) and capital letters (A/B) in the same row were used to indicate significant (*P* < 0.05) differences in isolation rates and MDR rates between production cycles 1 and 2, respectively.
